# Local autologous platelet rich plasma injection combined with platelet rich fibrin filling as the main treatment for refractory wounds: A case series

**DOI:** 10.3389/fsurg.2022.1003691

**Published:** 2023-01-09

**Authors:** Xiang Liu, Xiangjun Li, Wei Wei, Xiang Zhang, Zheng Fang, Zixiu Chen, Pingxiang Chen, Haihong Li

**Affiliations:** ^1^ Department of Wound Repair and Dermatologic Surgery, Taihe Hospital, Hubei University of Medicine, Shiyan, China; ^2^ Department of Wound Repair, Southern University of Science and Technology Hospital, Shenzhen, China

**Keywords:** platelet rich plasma, platelet rich fibrin, refractory wounds, autologous, wound repair

## Abstract

Refractory wounds are a major global health problem that not only affects the quality of life, but also causes significant physical, psychological and economic burdens. How to promote wound healing has become the main goal of clinicians. To evaluate the safety and efficacy of local autologous platelet rich plasma (PRP) injection combined with platelet rich fibrin (PRF) filling as the main treatment for refractory wounds. In the study, autologous PRP and PRF were prepared from whole blood. Twelve patients, each having a refractory wound, were included. The wounds were debrided first to remove necrotic and infected tissues, and then were given once or twice local PRP injection combined with PRF filling treatment. The total healing time (the time from admission to wound healing) and the healing time after PRP/PRF combined treatment (the time from PRP treatment to wound healing), as well as the adverse events were recorded. The results showed that the wound duration before the combination treatment was 10.48 ± 3.66 weeks, and the mean area/volume (for sinus or fistula) of the wounds was 8.23 ± 2.67 cm^2^/9.54 ± 2.13 cm^3^ (for sinus or fistula). All wounds healed after once or twice PRP/PRF combined treatment. The total healing time was 26.91 ± 8.01 days, and the healing time after the combined treatment was 16.36 ± 7.47 days. No adverse events were reported during the treatment and follow-up period. Our case series demonstrate the safety and synergistic effectiveness of local autologous PRP injection combined with PRF filling as the main treatment for refractory wounds. Platelet concentrates is not only an adjuvant treatment for chronic wounds, but a potential substitute for chronic wounds, especially in sinuses and fistulas.

## Introduction

Chronic refractory wounds refer to wounds that have undergone evidence-based treatment for more than 4 weeks but have no tendency to heal (1). It is estimated that nearly 10% of the population will suffer from chronic wounds during their lifetime, and the wound-related mortality rate is as high as 2.5% (2). In the United States, approximately 5 to 7 million people are affected by chronic wounds each year, while in China, the number of people affected is about 10 times that of the United States (3, 4). Chronic refractory wounds not only affect the quality of life, but also bring substantial physical, psychological and economic burdens to patients and their families (5). With an aging population and rising rates of obesity and chronic disease, the incidence of chronic refractory wounds is expected to rise (5).

The goal of chronic wound treatment is to promote wound healing and prevent the related complications, such as amputation (6, 7). Conventional wound treatments include local treatment and systematic treatment. Local treatments include debridement, antibiotic ointments, growth factors, dressings, vacuum-assisted closure (VAC), and surgical intervention (6, 7). Systemic treatments include anti-infection, removal or correction of causative factors, and treatment of comorbidities (6, 7). However, there are still many wounds fail to heal after the conventional treatments. Therefore, it is necessary to develop additional advanced wound treatments to promote the healing of these wounds.

In recent years, platelet concentrates have received positive responses as an adjuvant therapy for acute and chronic wounds (8, 9). Platelet concentrates can stimulate the supraphysiological release of multiple bioactive factors, such as growth factors, cytokines, and chemokines, which act at various stages of the wound healing through endocrine, paracrine, and autocrine mechanisms. In addition, neutrophils and macrophages in platelet concentrates play important roles in fighting microorganisms and removing cellular debris (8, 9). However, there are few reports on platelet concentrate as the main treatment for chronic wounds. In the case series, we evaluate the safety and efficacy of local autologous PRP injection combined with PRF filling as the main treatment for refractory wounds.

## Methods

### Patients

From December 2019 to June 2021, Inpatients who met the inclusion criteria and had no diseases listed in the exclusion criteria were included in the study. The study was carried out in accordance with the Declaration of Hesinki and was approved by the Institutional Review Committee of Taihe Hospital. Informed consent was obtained from the patients.

**Inclusion criteria:** Individuals with a wound lasted at least 4 weeks without exposing bones, muscles, tendons or ligaments; wound volume (length × width × height) was between 4 and 10 cm^3^, or wound area (length × width) was between 4 and 10 cm^2^.

**Exclusion criteria:** Individuals with cancer, connective-tissue disease, blood system disease, immunodeficiency disorder, mental disorder, severe cardiovascular disease and infection, and platelet count <100 × 10^6^/L, were excluded.

### Preparation of PRP and PRF

Autologous PRP and PRF were prepared from whole blood. According to the size of the wound, about 40 ml to 80 ml of antecubital venous blood was drawn from each patient.

PRF was prepared as follows. About 20–60 ml of venous blood without anticoagulant was immediately centrifuged at 3,000 revolutions per minute (rpm) for 10 min using a tabletop centrifuge machine at room temperature. The centrifuged whole blood was divided into three layers, the upper platelet-poor plasma (PPP) layer, the bottom red blood cell (RBC) clot layer, and the middle PRF clot layer. The PRF clot and the RBC clot were removed together from the centrifuge tube with sterile forceps, and then the RBC clot was separated from PRF clot using sterile scissors. The PRF clot was compressed between two pieces of gauze to obtain PRF plug, which was used as filling materials for the tissue defects.

PRP was prepared by a two-stage centrifugations process. About 20–40 ml of venous blood was drawn into sterile centrifuge tubes containing the anticoagulant 3.8% sodium citrate (anticoagulant: whole blood = 1:9). Blood and anticoagulant were thoroughly mixed to prevent formation of blood clots. 1 ml aliquot of whole blood was segregated and analyzed for pre-processed blood cell counts. After the whole blood sample was centrifuged at 900 × g (1700 rpm) for 5 min at room temperature, the blood was divided into three layers, the upper PPP layer, the middle intermediate buffy coat layer, and the bottom RBC layer. Then the upper layer and the middle layer were aspirated to another sterile centrifuge tube and was centrifuged at 1,500 × g (3000 rpm) for 15 min at room temperature. After the second centrifugation, the blood was divided into two layers, the upper PPP layer and the bottom platelet pellet layer. Platelet pellets was suspended in a quantity of PRP (8 ml whole blood prepared 1 ml PRP). 1 ml aliquot was separated for post-processed blood cell counts. The remaining PRP was aspirated to the sterile syringe for injection.

### Wound treatment and assessment

The refractory wounds were first debrided to remove necrotic and infected tissues, and then were flushed with normal saline, 3% hydrogen peroxide solution saline and povidone iodine. When the wounds were clean and fresh, autologous PRP combined with PRF was applied to the wounds. Based on the wound size and volume, 2–4 ml of PRP was injected into the wound beds and the subcutaneous tissue surrounding the wounds. PRF plug was filled into the fistula or sinus, or directly applied to the wound surface. The wounds were usually sutured with silk thread intermittently to prevent PRF plug from moving and promote wound healing. The wound surface was covered with PPP-soaked gauzes and finally bandaged. The dressing was changed first on the 3rd day post-treatment, and then every other day. If the wound did not heal within 10 days after the first combined treatment, a second combined treatment was given. The patients were followed up for 12 weeks post-treatment. Wounds were photographed, and the total wound healing time, the wound healing time after the combined treatment of PRP and PRF, and the adverse effects during the treatment were recorded. The total wound healing time was the time from the patient's admission to the complete healing of the wound. The wound healing time after the combined treatment of PRP and PRF referred to the time from the combined treatment to the complete healing of the wound.

### Statistical analyses

Data were analyzed using standard statistical software (SPSS 12.0) and Analysis ToolPak in Microsoft Excel. The values were expressed as mean ± standard deviation (SD). The statistical significance of values among groups was evaluated by the Student's *t* test. The difference was considered significant when the *P* value was 0.05 or less.

## Results

### General data of the included patients

**Figure 1:**
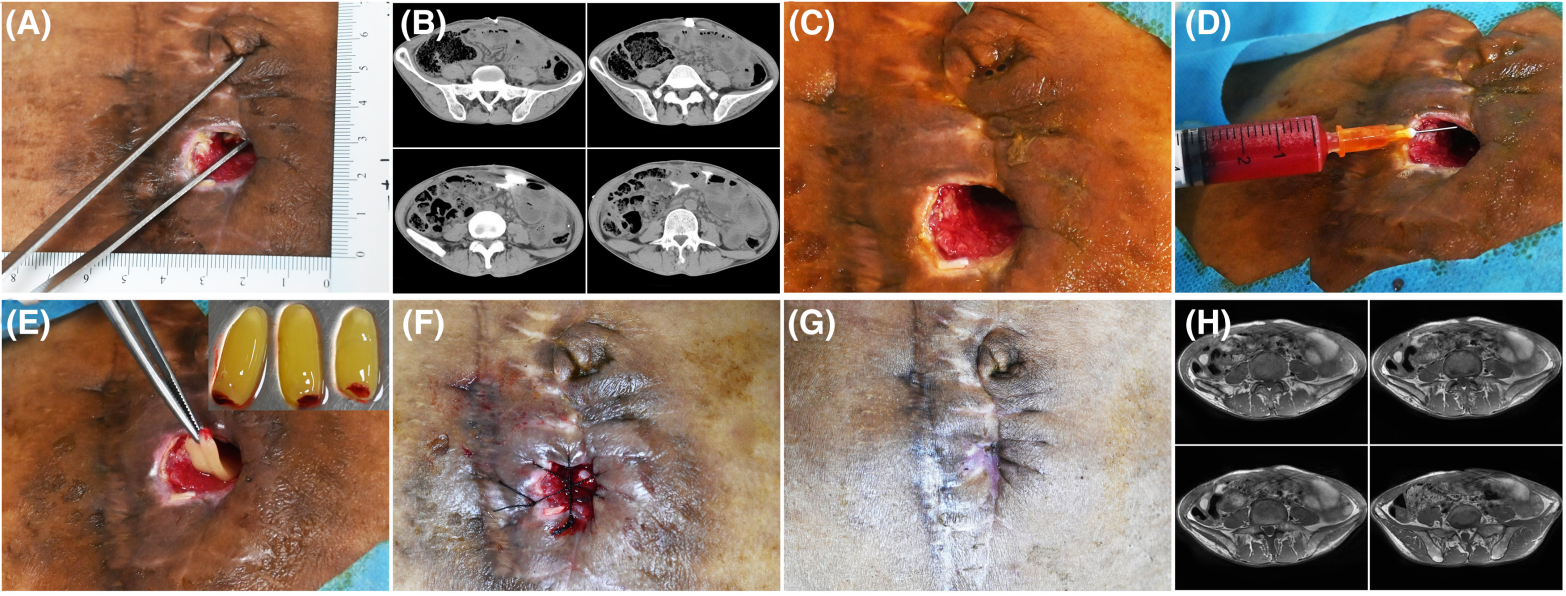
Patient 1. A 50-year-old man developed an abdominal fistula after intestinal adhesion lysis for 2 months. (**A**) Baseline. The depth of the sinus tract was checked with sterile surgical forceps. (**B**) The enhanced computed tomography showing the abdominal fistula. (**C**) Pre-PRP treatment. The wound was clean after debridement. (**D**) Treatment day. The wound bed was injected with PRP. (**E**) Treatment day. The fistula was filled with PRF. (**F**) Treatment day. The wound was sutured with silk thread. (**G**) 14 days after the treatment. (**H**) 16 days after the treatment, MRI showing the healing of abdominal fistula.

**Figure 2:**
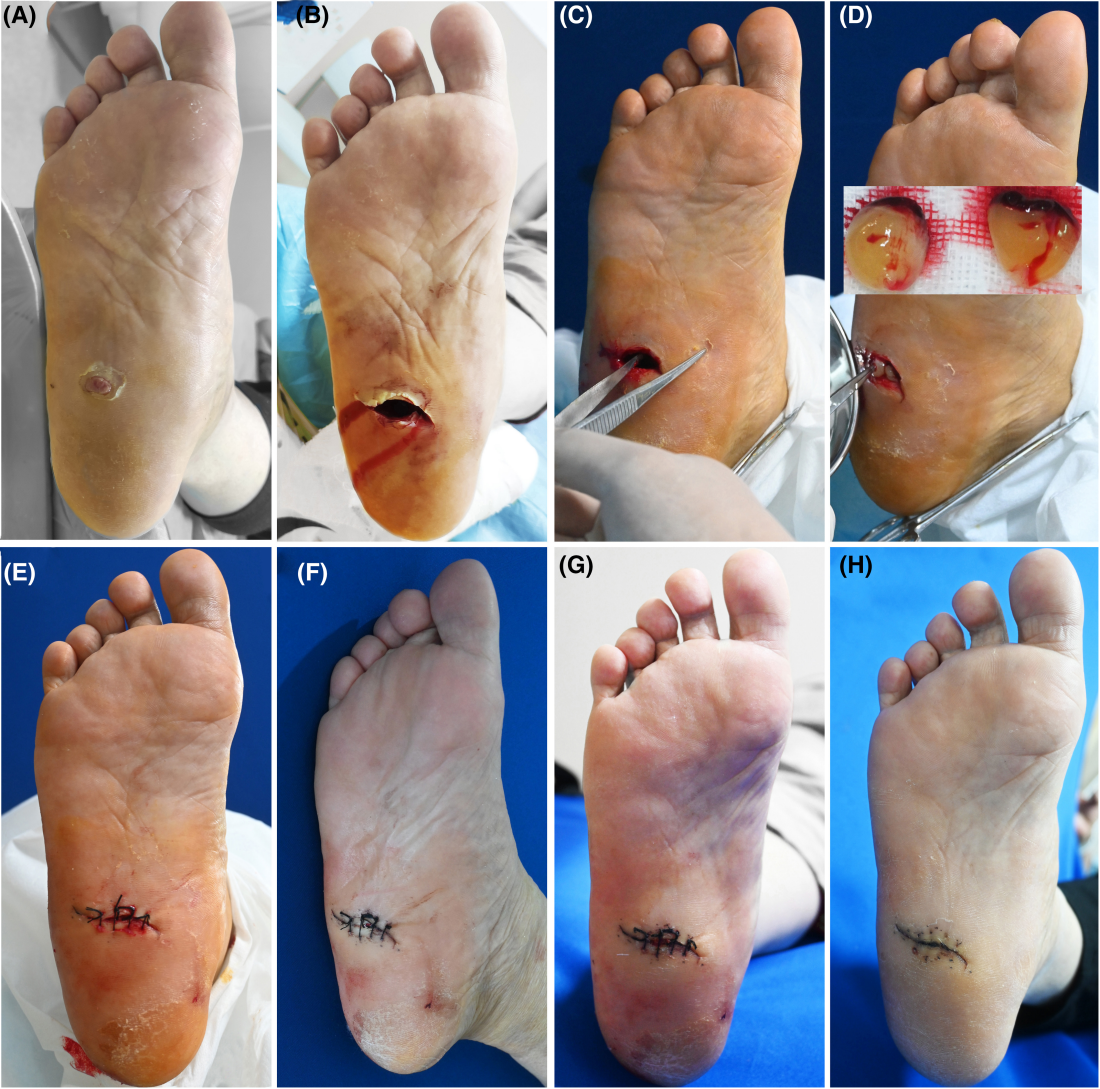
Patient 2. A 57-year-old female was stabbed by bamboo stubble on her right plantar for more than 2 months. (**A**) Baseline. (**B**) Sinus tract left after removal of the hyperproliferative granulation tissue. (**C**) Treatment day. The depth of the sinus tract was checked with sterile surgical forceps. (**D**) Treatment day. Prepared PRF. The tract was filled with PRF. (**E**) Treatment day. The wound was sutured with silk thread. (**F**) 3 days after the treatment. (**G**) 6 days after the treatment. (**H**) 21 days after the treatment.

**Figure 3:**
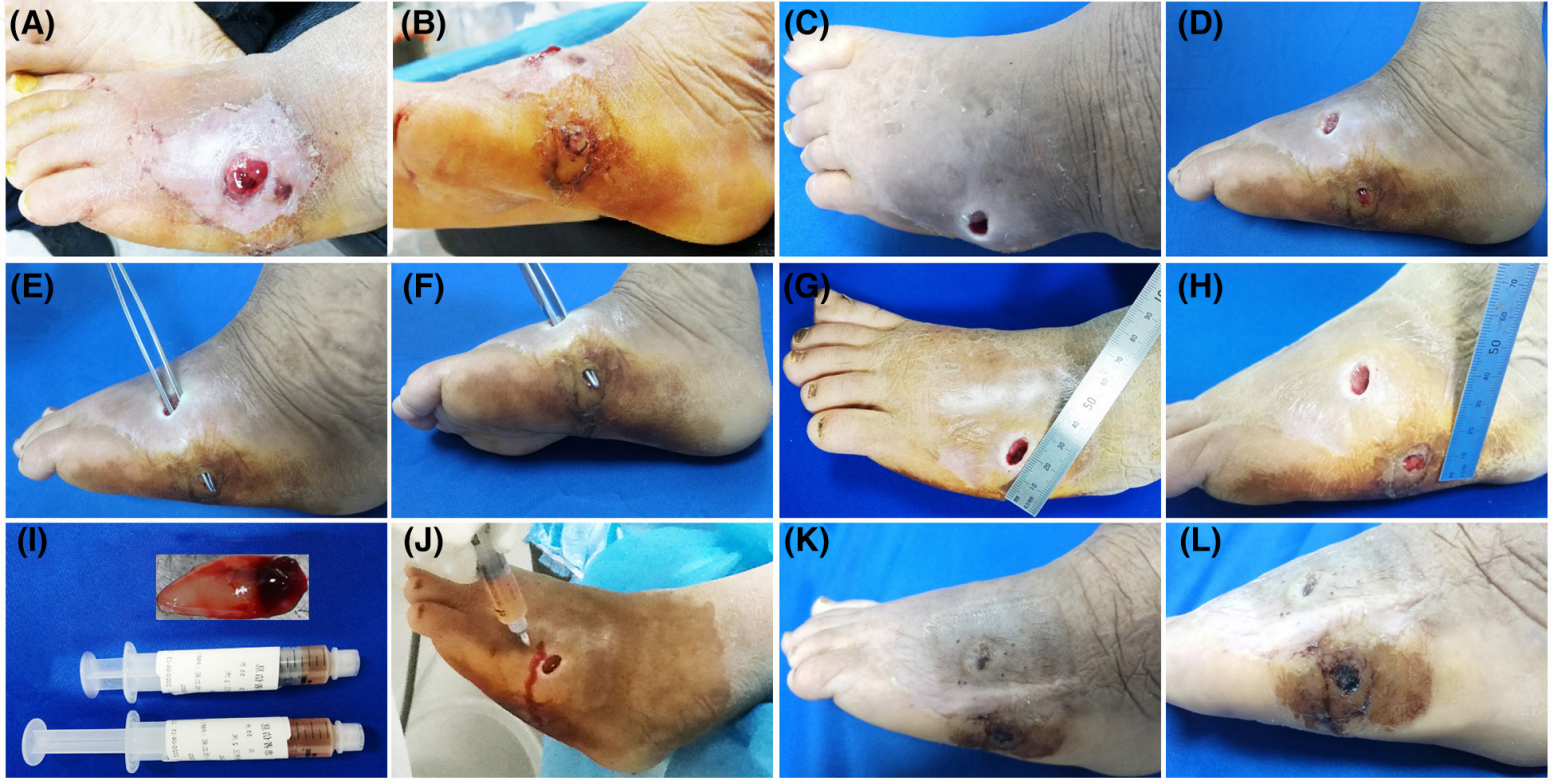
Patient 3. A 55-year-old female developed a fistula in the left lateral foot for 8 months. (**A–D**) Baseline. Diabetic foot, Wagner grade 2. (**E–H**) The wound was clean after debridement. The fistula was explored with sterile surgical forceps. (**I**) Treatment day. The prepared PRP and PRF. (**J**) Treatment day. The wound bed was injected with PRP. (**K,L**) 28 days after the combined treatment.

**Figure 4:**
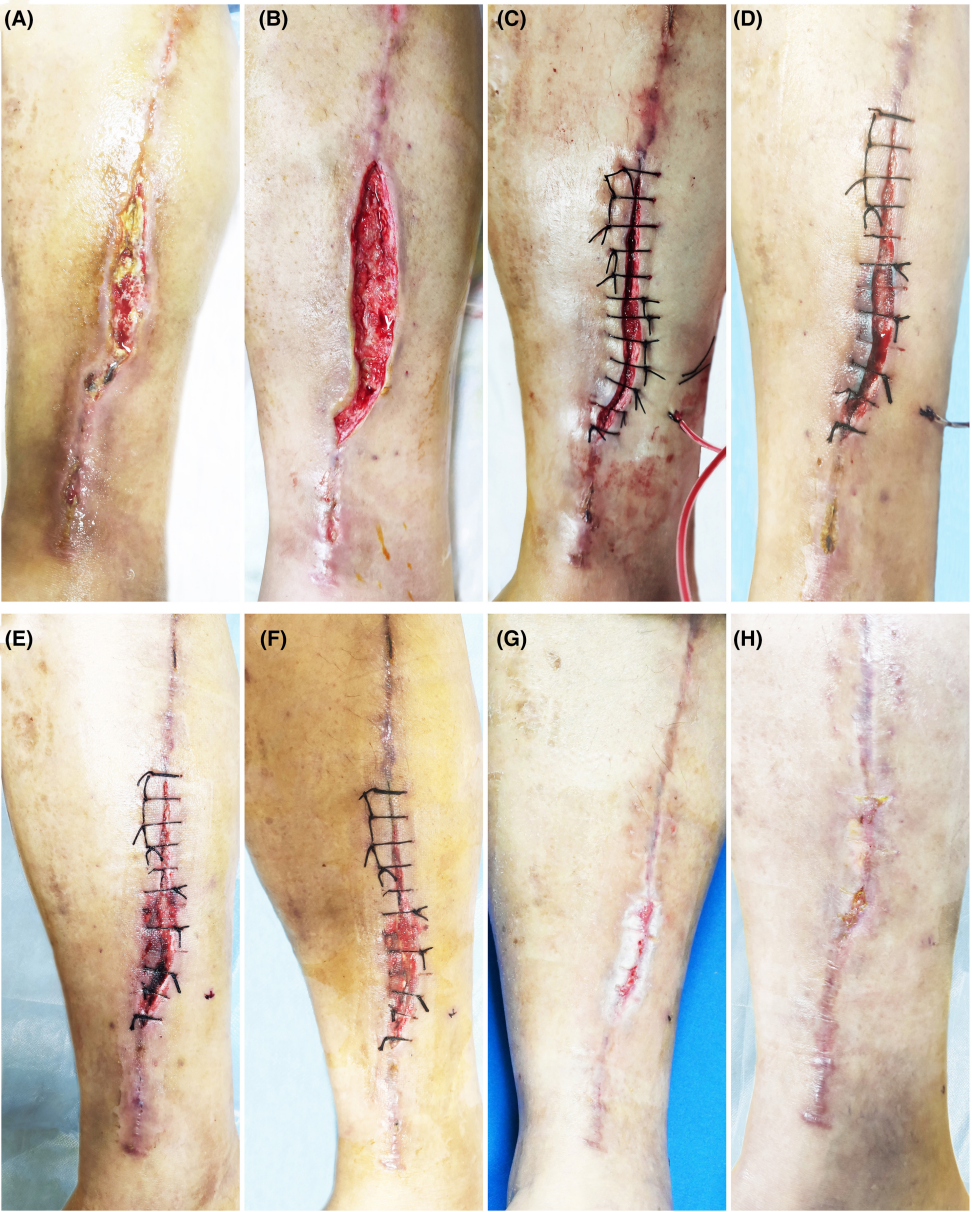
Patient 4. A 52-year-old male suffered a chronic wound on the medial side of his lower leg after undergoing a great saphenous vein graft*.* (**A**) Baseline. (**B**) Treatment day. The wound was clean after debridement. (**C**) Treatment day. The wound was sutured with silk thread, and then PRP was injected into the wound bed and the PRF was filled in the wound. (**D**) The second day after the treatment. (**E**) 3 days after the treatment. (**F**) 7 days after the treatment. (**G**) 14 days after the treatment. (**H**) 3 months after the treatment.

Twelve patients, 4 males and 8 females, in our department with refractory wounds were included in the study. The age ranged from 49 to 75 years old, with an average of 58.50 ± 8.09 years old. The wound duration before the combined treatment varied from 4 weeks to 32 weeks, with an average of 10.48 ± 3.66 weeks. The mean area/volume (for sinus or fistula) of the wounds was 8.23 ± 2.67 cm^2^/9.54 ± 2.13 cm^3^ (for sinus or fistula). The wounds occurred in various parts of the body, including 7 cases in the feet (Figures 2, 3), 3 cases in the legs (Figure 4), 1 case in the temporal region, and 1 case in the abdomen (Figure 1). The etiologies were various also, including 6 cases of diabetic foot wound (Figures 2, 3), 3 cases of vascular wound, 2 cases of iatrogenic wound (Figures 1, 4), and 1 case of pressure wound.

### Blood cell concentration in the whole blood and the prepared PRP

The platelet concentration increased from 206.75 ± 52.37 × 10^9^/L in the whole blood sample to 891.78 ± 82.95 × 10^9^/L in the prepared PRP sample. The white blood cell (WBC) concentration in the prepared PRP was 5.06 ± 1.68 × 10^9^/L, which was slightly higher than the whole blood WBC concentration of 4.82 ± 1.57 × 10^9^/L. Compared with the whole blood red blood cell (RBC) concentration of 3.62 ± 0.99 × 10^12^/L, the RBC concentration in the prepared PRP was significantly reduced to 0.19 ± 0.08 × 10^12^/L.

### Efficacy and safety of the combined treatment of PRP and PRF

All the patients showed wound healing (Figures 1–4). Nine patients received the combination treatment of PRP and PRF once, and three patients received the combination treatment twice. The average total healing time was 26.91 ± 8.01 days, and the average healing time after PRP and PRF combined treatment was 16.36 ± 7.47 days. Before the combined treatment of PRP and PRF, it took approximately 9.27 ± 3.27 days to clean the wounds. The patient tolerated the treatment procedure well. On the day of treatment and during the follow-up period, all patients had no discomfort.

## Discussion

Wound healing is a highly dynamic process involving complex interactions of extracellular matrix, various growth factors and cytokines, resident cells and blood cells (1, 9). These interactions must be closely coordinated to achieve tissue integrity and homeostasis. If the interaction is interrupted or uncoordinated, chronic wounds will be formed (1, 9). Chronic wounds are often difficult to treat because wounds with impaired healing are not easy to restart. VAC, skin grafts, and flap grafts have shown good results in many but not all cases. Platelet concentrates contain a variety of cells, growth factors and cytokines that provide an internal biological environment to support the three stages of the wound healing cascade: inflammation, proliferation and remodeling. These properties make platelet concentrates an alternative treatment to promote wound healing.

In our case series, 12 refractory wounds were treated with a combination of local autologous PRP injection and PRF filling. The wounds healed completely, and the mean healing time was 26.91 ± 8.01 days. In a study of 13 wounds treated with PRF (mean duration of treatment 4.2 weeks), 8 wounds were closed, 3 wounds had a 66% reduction in diameter, 1 wound had a reduction in depth but not size, and 1 wound had no change (10). Another study of 24 wounds showed that the size of 17 wounds was reduced by 90%, and 3 wounds was reduced by 80%–90% after PRP treatment (9). A recent meta-analysis of PRP in chronic refractory wounds, including seventeen randomized controlled trials, showed that compared with the control group, PRP significantly increased the percentage of healed wounds and the percentage of the healed area (11). All the studies showed the effectiveness of PRP and PRF in the treatment of chronic wounds. Autologous PRP was much safer than allogeneic PRP, although they were both effective in chronic refractory wounds (11). In this study, the combined use of autologous PRP injection and PRF filling resulted in a better wound healing rate than the use of PRP and PRF alone reported in the literature.

The prepared PRF was solid, so we did not count the concentration of blood cells in it. We only counted and compared the concentration of blood cells in whole blood and PRP. The platelet concentration in PRP was more than 4 times the baseline value of whole peripheral blood, and the concentration of WBCs in the prepared PRP was slightly higher than that of whole blood, but the concentration of RBCs in PRP was significantly lower than that of whole blood, which was about one-twentieth of that of whole blood. Although the dependence of clinical effectiveness on the platelet concentration in PRP was inconsistent and there are few precise recommendations regarding PRP concentration, it has been suggested that the platelet concentration in PRP to achieve a therapeutic effect should be 3 to 5 times the baseline (12). Most researchers believe that PRP and its derivatives contain an appropriate number of WBCs can prevent infections and promote wound debridement, but the presence of RBCs in PRP usually produces detrimental and cytotoxic effects through the production of pro-inflammatory mediators, oxidative stress, and immunosuppression (13). The PRP prepared in our study meets the prerequisites for PRP to produce significant clinical therapeutic effects, such as platelet dosage, minimal RBC contamination and the presence of WBC (12). The activation of PRP in our study is not through the addition of exogenous activators, but the endogenous tissue damage caused by multi-point injection, which is in line with the “minimal handling product” considered by the Food and Drug Administration (FDA) of the United States (14).

The combined use of PRP and PRF has synergistic effects in promoting chronic wound healing. PRP is the first-generation platelet concentrate, and PRF is the second-generation platelet concentrate, which is emerged as a more advanced PRP (15). Both PRP and PRF can stimulate the supraphysiological release of various growth factors and cytokines to accelerate soft-tissue healing and restore tissue homeostasis, but they have different release kinetics. PRP released higher growth factors at an earlier time point, whereas PRF released growth factors continuously and steadily (16). The total protein content of platelet derived growth factor (PDGF)-BB and vascular endothelial growth factor (VEGF) in PRP was significantly higher than that of PRF, and the total protein content of PDGF-AA, PDGF-AB, epidermal growth factor (EGF) and insulin-like growth factor (IGF) in PRF was significantly higher than that of PRP over a 10-day period (16). Different growth factors play different roles in the wound repair cascade (12). Moreover, PRP provides a suitable microenvironment and PRF provides a fibrin matrix, and they synergistically promote cell proliferation and differentiation, granulation tissue formation, angiogenesis, and tissue regeneration.

In conclusion, this case series demonstrates the safety and synergistic efficacy of topical autologous PRP injection combined with PRF filling as the primary treatment for chronic refractory wounds, especially sinuses and fistulas with a volume of less than 10 cm^3^. PRP/PRF is not only an adjuvant treatment for chronic wounds, but also a potential main treatment for chronic wounds.

## Data Availability

The original contributions presented in the study are included in the article/Supplementary Material, further inquiries can be directed to the corresponding author/s.
